# Some Recent Advances in Physiology and Pathology

**Published:** 1905-05-13

**Authors:** 


### Hospital Clinics.

### SOME RECENT ADVANCES IN PHYSIOLOGY AND PATHOLOGY.

Dr. H. Batty Shaw[1] deserves well of all busy practitioners who desire to march with the times for his laborious and interesting collection of recent additions to our knowledge of the secretory tissues. A brief summary of some of the more important items will probably be welcome to a good many who have an uneasy consciousness that the physiological mechanisms of the human body are not quite what they were in student-days, and who yet lack full opportunity to master the changes.

*The Stomach.*—Bolton, investigating the causation of gastric ulcers, has found that by injecting into an animal (B) an emulsion of stomach-cells from a different animal (A), a serum can be obtained which has a specific erosive action upon the stomachs of animals of the same species as A. Thus an emulsion of the stomach-cells of a guinea-pig injected into the peritoneal cavity of a rabbit produces in the rabbit a serum which, while harmless to other rabbits, yet when injected into a guinea-pig rapidly produces multiple erosions of the gastric mucosa.

*The Intestine and Pancreas.*—Much has been learnt about the relation of the pancreatic secretion and the succus entericus. The course of intestinal digestion is now believed to be the following:—The acid gastric-juice passing into the small intestine acts upon a body present in the mucosa of this part of the gut and known as pro-secretin, converting it into secretin. This secretin, being absorbed by the blood-stream, on reaching the pancreas stimulates the cells of this organ to the production of an inactive substance called trypsinogen. This body is made active by an ingredient of the succus entericus called enterokinase, and whereas it was previously practically inert towards proteids, it now becomes actively proteolytic. The succus entericus contains another important ferment called erepsin, which, though inert towards albumin, is capable of further splitting up albumoses and peptones, and thus preparing them for absorption. It has been proved that pancreatic juice taken from the pancreatic duct of man is incapable, when pure, of digesting proteids, though it may be activated by succus entericus taken from a dead body. On the other hand, succus entericus derived from a dog is incapable of activating pure human pancreatic juice, "an important observation in view of the attempts made to employ the succus entericus of lower animals when such secretion is deficient in man." But the property of activating pancreatic juice is not limited to the intestinal secretion; it resides in the lymphatic glands and in the leucocytes of exudates and of the blood, and fibrin is slowly digested by inactive pancreatic juice, thus proving the existence in itself of an activating substance or kinase.

A greater importance is becoming attached to the digestive capabilities of bacteria. It has been found that they are not only able to digest proteids themselves, but that they, and even the filtrates of cultures, are able to activate inactive juices.

The potentialities of these results are very striking, and stimulating to the imagination. Dr. Shaw says: "In following these accounts of recent investigations the reader is struck by the probable changes that will take place in medicine; an intimate knowledge will be required of unorganised ferments. The study of animal and vegetable cells is greatly broadened by these and other investigations on their physiology, and it would appear that bacteria, which have long held the field as agents exercising a beneficial or harmful influence on the economy of the body, must share this position with the unorganised ferments derived from secretory cells independently of mycotic action. In this way light may be thrown in the near future upon a number of obscure disorders which for want of a better title are described as toxic in origin, though what is the nature of these toxic bodies, and how they are produced, is an entire enigma; whilst the ravages they produce are amongst the most familiar experiences in pathological study, such as the degeneration of sensory axons in tabes dorsalis, pernicious anæmia, tetany, diabetes mellitus, etc."

*The Testicle.*—Many attempts have been made to establish the existence of an internal secretion of the testicle, but no definite results have been achieved. Careful observation of castrated rabbits shows that the result of this deprivation is an enlargement of the thyroid gland and of the medullary portions of the supra-renal bodies. No change was found in the parathyroids, thymus or pituitary bodies.

The function of the *pituitary body* still defies definition, and opinions as to its relation to acromegaly are very discordant.

[1] Pract., April 1905.

*(To be continued.)*

